# Dominant-negative VPS-4 disrupts ODR-10::GFP distribution but has limited effects on chemotaxis

**DOI:** 10.17912/gnyw-v322

**Published:** 2018-08-02

**Authors:** Ellen Zocher, Nelson Ruth, Caroline Dahlberg

**Affiliations:** 1 Biology Department, Western Washington University, Bellingham, WA 98225

**Figure 1.**
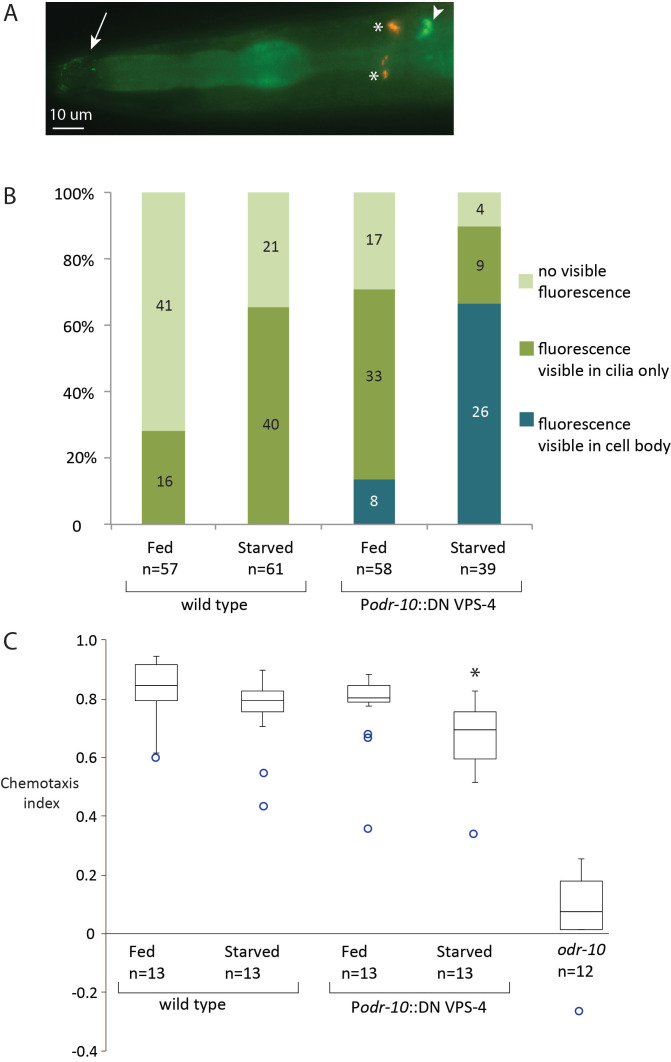
Effects of expression of *vps-4(E219Q)* (VPS-4 DN) under the *odr-10* promoter. A. Image of the head of an animal to illustrate the distribution of ODR-10::GFP fluorescence in an AWA neuron of an animal expressing VPS-4 DN under the *odr-10* promoter. Animals were scored for having no visible fluorescence, fluorescence in the cilia, fluorescence in the cell body (arrowhead), with or without ciliary fluorescence (arrow). Red fluorescence is from the injection marker, *Pttx-3*::dsRed (asterisks). Image acquired at 630X magnification. B. Localization of ODR-10::GFP in the AWA neurons of adult hermaphrodites. Bar graph shows the relative distribution of phenotypes under different conditions. Numbers in the columns indicate the number of animals observed. Data are significant with p << 0.001 using a Chi-square contingency test, Chi-Square value = 123.82, 6 degrees of freedom. No assumptions were violated. C. Effects of different conditions on chemotaxis toward diacetyl, compared to ethanol control for young adult hermaphrodites; *odr-10* (*ky32*) is shown as a negative control. Animals in chemotaxis experiments did not express ODR-10::GFP. Box and whisker plots show the results of 12-13 independent trials. Median chemotaxis indices were wild type (N2) = 0.85, wild type (N2), starved = 0.8, VPS-4 DN = 0.81, VPS-4 DN, starved = 0.7, *odr-10* (*ky32*) = 0.08. Boxes bound the 1st and 3rd quartiles and circles represent outliers of greater than 1.5 times either quartile. Chemotaxis indices were calculated using the following expression: ((animals at chemoattractant)-(animals at control))/total animals on the plate. * p<0.05 compared to wild type, Tukey-Kramer post-test.

## Description

Accumulation of ODR-10 depends on the sex and fed state of *C. elegans* (Ryan et al. 2014), though the precise mechanism of its regulation is not known. We tested whether the ESCRT pathway is required to maintain normal accumulation and distribution of ODR-10::GFP in AWA neurons under well-fed and starved conditions. The conserved AAA ATPase VPS-4 is required as the final step of the ESCRT pathway, to dissociate the machinery as cargo is moved from endosomes to multivesicular bodies (Babst et al. 2011, Kim et al. 2011). Mutation of Glutamate 219 to Glutamine (E219Q) in VPS-4 results in a dominant negative enzyme (VPS-4 DN). VPS-4 DN disrupts normal accumulation of the glutamate receptor, GLR-1, in a cell autonomous manner (Chun et al. 2008).

We investigated a potential role for VPS-4 in ODR-10 regulation using fluorescence microscopy and an attraction chemotaxis assay. We expressed VPS-4 DN under the *odr-10* promoter in animals expressing ODR-10::GFP (Figure 1A). Using live-imaging (200X (total) magnification), ODR-10::GFP was not visible in over half of the wild type, well-fed animals. Using this baseline, we measured changes in ODR-10::GFP (abundance and localization) and found that VPS-4 DN increased the frequency with which we observed animals with aberrant ODR-10 accumulation (Figure 1B). In 8 animals (out of 58) there was observable fluorescence in the cell bodies of AWA neurons (1 with fluorescence exclusively in the cell body). Starvation exacerbated this effect: 26 out of 39 animals had fluorescence in the cell bodies of AWA neurons (7 with fluorescence exclusively in the cell body). In contrast to previously published data that relied on different imaging parameters (Ryan, et al., 2014) we also observed that starvation of animals led to increased frequency of visible fluorescence in the cilia of AWA in WT animals (Figure 1B). Expression of VPS-4 DN had no effect on the ability of animals to react to the chemoattractant diacetyl under well-fed conditions (Figure 1C). However, VPS-4 DN expression in the AWA neuron resulted in significantly lower chemotaxis indices, compared to wild type, fed animals (Figure 1C, asterisk).

Starvation of animals has a subtle effect on the abundance of ODR-10 that accumulates in the cilia of AWA neurons, but that accumulation does not affect the efficiency of chemotaxis in wild type animals (Figure 1B and C). However, when food stress was paired with a disruption of the ESCRT pathway the aberrant accumulation of ODR-10::GFP correlated with a modest but significant decrease in chemotaxis index (Figure 1C). It is still unknown whether ODR-10 is a direct target of the ESCRT machinery or other ubiquitin-related pathways. These data could also represent indirect effects due to changes in availability or localization of cofactors that are required for normal receptor processing and/or localization within the AWA neuron.

## Methods

Animals were reared on lawns of OP50 on NGM plates according to established protocols. Well-fed, uncrowded young adult hermaphrodites were used for both imaging and chemotaxis assays. For assays using “starved” animals, L4 larvae were moved to NGM plates lacking bacteria 18-24 hours prior to assay. For fluorescence microscopy, young adult hermaphrodites were placed in 3µl M9 on coverslip, which was then inverted onto 3µl of solidified 2% agarose with 0.5% Levamisole (MP Biomedicals, Inc.) on the microscope slide. Animals were imaged on a Leica DMi6000 inverted microscope equipped with a Leica DFC3000G cooled CCD camera and GFP filter, at 200X total magnification (20X objective, NA 0.4). Animals were imaged using 150 ms exposures and gain of 10. Animals were described as having “no fluorescence” if no cells could be detected using these imaging parameters. Chemotaxis was assayed as previously published (Sengupta et al. 1996) using young adult hermaphrodites, 0.1% (v/v) diacetyl in ethanol was used as the attractant and ethanol as a negative control, with 0.02% (w/v) sodium azide as a paralytic at the site of the chemoattractant and control. Animals used for chemotaxis assays did not express ODR-10::GFP.

## Reagents

Strains: N2 (Bristol) CX3344 *kyIs53* [*Podr-10::*ODR-10*::GFP*] CLD5 *dahEx5* [*Podr-10::*VPS-4 DN (E219Q)*; Pttx-3::dsRed*]; *kyIs53*CLD17 *dahEx5; kyIs53*CX32 *odr-10(ky32)*

## References

[R1] Ryan DA, Miller RM, Lee K, Neal SJ, Fagan KA, Sengupta P, Portman DS (2014). Sex, age, and hunger regulate behavioral prioritization through dynamic modulation of chemoreceptor expression.. Curr Biol.

[R2] Babst M, Davies BA, Katzmann DJ (2011). Regulation of Vps4 during MVB sorting and cytokinesis.. Traffic.

[R3] Chun DK, McEwen JM, Burbea M, Kaplan JM (2008). UNC-108/Rab2 regulates postendocytic trafficking in Caenorhabditis elegans.. Mol Biol Cell.

[R4] Kim DW, Sung H, Shin D, Shen H, Ahnn J, Lee SK, Lee S (2011). Differential physiological roles of ESCRT complexes in Caenorhabditis elegans.. Mol Cells.

[R5] Kowalski JR, Dahlberg CL, Juo P (2011). The deubiquitinating enzyme USP-46 negatively regulates the degradation of glutamate receptors to control their abundance in the ventral nerve cord of Caenorhabditis elegans.. J Neurosci.

[R6] Sengupta P, Chou JH, Bargmann CI (1996). odr-10 encodes a seven transmembrane domain olfactory receptor required for responses to the odorant diacetyl.. Cell.

